# *Helicobacter* Species and Their Association with Gastric Pathology in a Cohort of Dogs with Chronic Gastrointestinal Signs

**DOI:** 10.3390/ani12101254

**Published:** 2022-05-13

**Authors:** Roman Husnik, Jiri Klimes, Simona Kovarikova, Michal Kolorz

**Affiliations:** 1 Department of Veterinary Clinical Sciences, College of Veterinary Medicine, Purdue University, 625 Harrison Street, West Lafayette, IN 47907, USA; 2 Department of Biology and Wildlife Diseases, Faculty of Veterinary Hygiene and Ecology, University of Veterinary Sciences Brno, Palackeho 1/3, 612 42 Brno, Czech Republic; mvdr.klimes@gmail.com; 3 Department of Animal Protection, Welfare and Behavior, Faculty of Veterinary Hygiene and Ecology, University of Veterinary Sciences Brno, Palackeho 1/3, 612 42 Brno, Czech Republic; kovarikovas@vfu.cz; 4 Department of Clinical Pharmacy, University Hospital Martin, 036 01 Martin, Slovakia; michalkolorz@seznam.cz

**Keywords:** chronic gastritis, non-*Helicobacter pylori* helicobacters, PCR, urease test, cytology

## Abstract

**Simple Summary:**

*Helicobacter* spp. represent spiral-shaped Gram-negative bacteria that can live in the acidic environment of the stomach. While their association with peptic ulcers and gastric neoplasia in people has been clearly documented, their pathogenic potential in dogs is less well defined. *Helicobacter pylori*, the most prevalent *Helicobacter* infecting people, does not seem to represent a significant problem in small animals. The aims of this study were to determine the prevalence of individual gastric *Helicobacter* species in dogs with chronic gastrointestinal signs, their association with gastric lesions, and to compare diagnostic techniques used to detect *Helicobacter* infection in dogs.

**Abstract:**

Prevalence of individual *Helicobacter* species, data evaluating their association with gastric pathology and comparison of accuracy of diagnostic techniques are limited. The aims of this study were to determine the prevalence of gastric *Helicobacter* species, their association with gastric pathology, and to compare diagnostic techniques. Gastric biopsies from 84 privately-owned dogs with chronic gastrointestinal signs were obtained endoscopically. Helicobacters were detected using PCR, cytology, urease test, and histopathology. PCR detected helicobacters in 71.4% of dogs. *Helicobacter heilmannii* sensu stricto (s.s.) was the predominant species. Mixed infection was detected in 40% of PCR positive dogs. Gastritis was diagnosed in 38.5% of *Helicobacter* positive and 47.4% of *Helicobacter* negative dogs. Mono-infection was associated with 2.4 times increased odds of having more severe inflammation compared to mixed infection. Erosions and ulcers were common endoscopic lesions. Cytology had sensitivity/specificity of 88.3/91.7%. Association between infection and lymphoid follicular hyperplasia was demonstrated.

## 1. Introduction

In humans and several animal species helicobacters have been recognized to cause chronic gastritis, gastric ulceration, and gastric neoplasia [1,2,3,4]. *Helicobacter* species other than *H. pylori* were initially referred to as “*Gastrospirillum hominis*” but were later reclassified under the provisional name “*Helicobacter heilmannii*”. Further genetic analyses revealed several types of “*H. heilmannii*” which in turn proved to be a group of species later described as *H. suis*, *H. felis*, *H. bizzozeronii*, *H. salomonis*, and “*Candidatus H. heilmannii*” [5,6]. This organism was cultivated from feline gastric mucosa and described as a valid novel species *H. heilmannii* [7]. The name *Helicobacter heilmannii* sensu stricto was proposed to distinguish it from *Helicobacter heilmannii* sensu lato of earlier studies [8]. A closely related species was isolated from a dog and described as *H. cynogastricus* [6]. Except for *H. suis*, all the above-mentioned species other than *H. pylori* have been found in dogs. The gastric spiral bacteria that do not resemble *H. pylori* should be collectively referred to as gastric “non-*Helicobacter pylori* helicobacters” (NHPH) [5,6]. The aforementioned changes in the taxonomy and nomenclature as well as the difficulty to cultivate *in vitro*, isolate and identify the species have caused confusion in the description of NHPH infections in both human and veterinary medicine [9,10,11]. In clinical practice, *Helicobacter* infection in dogs has usually been diagnosed by microscopy of cytology specimens, rapid urease test of a gastric mucosal sample, or histologic evaluation of biopsies [12]. However, none of these methods enables the species determination. Species identification usually relies on PCR using genus-specific and species-specific primers [6].

The pathogenic role of NHPH in dogs has not yet been convincingly documented [13]. Almost all dogs are naturally infected; therefore, it is difficult to find negative control animals for conclusive studies [14]. The pathologic changes are generally milder in dogs than in humans with the NHPH infection. Helicobacters are encountered at a similar prevalence in dogs with clinical signs of gastrointestinal (GI) disease, gastritis, and in normal dogs [15,16]. The improvement or resolution of clinical signs following treatment suggests a possible causal relationship between these bacteria and canine gastritis [17]. However, the gastric histological lesions usually persist even after clinically successful treatment [18,19]. The development of gastritis and vomiting in some infected dogs has been attributed to the loss of host tolerance to gastric NHPH rather than to the pathogenicity of NHPH [20]. Precise knowledge about the prevalence of individual *Helicobacter* species is essential to solve the questions concerning the pathogenicity and zoonotic potential of canine gastric helicobacters [21,22].

Since the prevalence of individual Helicobacter species may differ geographically [23,24,25], we conducted the present study to explore the situation in dogs in the Czech Republic. The aim of this study was to identify the prevalence of the individual *Helicobacter* species in a cohort of dogs with chronic GI signs from Central Europe, to evaluate the association of *Helicobacter* species with histologically confirmed gastritis, and to compare the accuracy of different diagnostic techniques.

## 2. Materials and Methods

### 2.1. Patient Selection, Clinical Evaluation

Privately owned dogs were prospectively enrolled in the study if they were referred for clinical work-up of chronic GI clinical signs (>3-week duration) after exclusion of non-GI diseases and GI foreign body between November 2010 and February 2012. Clinical signs included vomiting, diarrhea, anorexia, or weight loss. The minimal diagnostic evaluation included physical examination, complete blood count, serum biochemistry profile, urinalysis, fecal flotation, and abdominal ultrasonography. In some dogs, thoracic and/or abdominal radiographs, trypsin-like immunoreactivity, pancreatic lipase immunoreactivity, serum folate and cobalamin concentrations were performed alone or in some combination as deemed appropriate. All enrolled patients were fed a hypoallergenic diet (Hill’s Prescription Diet z/d Canine or Canine Hypoallergenic Dry Dog Food by Royal Canin) for at least 2 weeks and all medications were discontinued at least 2 weeks before the endoscopy. The study protocol was approved by the animal care committee of University of Veterinary Sciences Brno and informed consent was obtained from all dog owners.

### 2.2. Gastrointestinal Endoscopy and Sampling

Esophagogastroduodenoscopy was performed in each dog. Pediatric gastroscope (Olympus XP 20) for toy and small and colonoscope (Olympus CF 40L) for medium and large breed dogs were used. After a 12-h fast each dog underwent general anesthesia using routine protocols. Multiple gastric biopsy samples were collected from the fundus, corpus, and antrum (usually 5 from each area). After each procedure the endoscope and biopsy forceps were cleaned and sterilized using an activated aldehyde solution (Cidex OPA, Johnson & Johnson, Irvine, CA, USA).

### 2.3. Helicobacter Status and Evaluation of Mucosal Inflammation

*Helicobacter* infection was documented in the gastric mucosa using a PCR assay of gastric biopsies, rapid urease test, cytological and histopathologic examination. PCR was considered to be the reference test in this study.

At least one (1–2) gastric mucosal biopsy sample from each dog was used to perform a rapid urease test. Two drops of solvent were added into a detection tube with dehydrated substrate (Itest plus, Hradec Králové, Czech Republic). After complete dissolution, the biopsy sample was submerged in the solution. The medium was evaluated for color change 24 h after submersion of the biopsy sample. At least one (1–2) biopsy sample per dog was used for the preparation of impression smears. These cytology specimens were stained with Giemsa-Romanowski and evaluated for presence of gastric spiral organisms under a 100x oil immersion lens.

DNA was isolated from one to two gastric mucosal biopsies using a tissue DNA isolation kit (QIAamp DNA Mini Kit^®^DNA, Qiagen, Germantown, MD, USA) according to the manufacturer’s protocol. PCR and nested PCR were used for the amplification of genus and species-specific sequences. Previously reported primer sequences and PCR protocols were used with modifications [26,27,28,29,30]. Primer sequences, genes detected, and size of amplification products are listed in Table 1.

Primer sequence specificity was confirmed by comparison with DNA sequences of individual *Helicobacter* species available in the GenBank database. PCR conditions and reaction selectivity were optimized and verified using positive DNA controls of available *Helicobacter* species and by DNA sequencing. The amplification parameters are listed in Supplementary file S1. Reaction volume was 20.0 μL. All PCR reactions were performed with 1U HotStarTaq^®^ polymerase (HotStarTaq^®^ polymerase, Qiagen, Germantown, MD, USA) and 0.1 μL of primers (100 pmol/μL). Products of amplification were analyzed by electrophoresis on a 2% agarose gel (Agarose, Amresco, Solon, OH, USA) and visualized with ethidium bromide under UV light.

The remaining gastric biopsies were prepared and processed for histopathological examination, fixed in buffered 10% neutral formalin, dehydrated, embedded in paraffin wax, sectioned on a microtome at a thickness of 4 μm, and stained with hematoxylin and eosin (HE) or with silver impregnation using the Warthin-Starry stain (WS). All slides were reviewed by one pathologist. Histological lesions were evaluated and screened for the presence of spiral bacteria in the surface mucus, gastric glands, and parietal cells. The severity of inflammatory changes was graded using a published system with scores from 0 (absent inflammation) to 3 (severe inflammation) [31,32]. The association between *Helicobacter* infection status and inflammatory infiltration, lymphatic follicular hyperplasia, epithelial injury, and presence of fibrosis was assessed.

### 2.4. Statistical Analysis

The normality of data distribution was tested using the D’Agostino and Pearson omnibus normality test. Unless otherwise stated, data are reported as means and standard deviations. Student’s *t*-test was used to assess the difference in age and body weight of *Helicobacter* positive and negative dogs. Chi-squared test was used to assess the difference in sex distribution. The Fisher’s exact test was used to assess the association between *Helicobacter* infection status and any of the endoscopic findings, the presence of gastritis, epithelial injury, fibrosis, or lymphoid follicular hyperplasia and to evaluate the association between the presence of single *Helicobacter* species or mixed *Helicobacter* infection and degree of gastric inflammation. The proportional odds model was used to examine the effect of the presence of single or mixed infection on severity of gastric inflammation. Two statistical software programs were used as appropriate (GraphPad Prism, GraphPad Software Inc., La Jolla, CA, USA; JMP Statistical Discovery, SAS, Cary, NC, USA) and a value of *p* < 0.05 was considered significant.

## 3. Results

Demographic data of 84 dogs (both *Helicobacter* positive and negative) enrolled into the study is given in Table 2. There was no significant difference in age (*p* = 0.58), body weight (*p* = 0.61), or sex (*p* = 0.11) between the two groups.

PCR detected *Helicobacter* DNA in 60 dogs (71.4%). Cytology of impression smears was positive in 55 of 84 dogs (65.5%). The rapid urease test was positive in 53 of 84 dogs (63.1%). Histopathologic examination of gastric biopsies revealed helicobacter in 53 of 84 dogs (63.1%) visible with both HE and WS. Comparison of different diagnostic techniques and result combination patterns for the *Helicobacter* spp. infection in dogs are given in Table 3.

Using a combination of diagnostic tests for *Helicobacter* including rapid urease test, cytology, histopathology, and PCR, 65 dogs (77.4%) had one or more positive results. Forty-two dogs (50%) were positive for all tests performed while 19 dogs (22.6%) were negative. Thus, the various diagnostic tests were concordant in 61 dogs (72.6%). Comparison of the diagnostic value of cytology, rapid urease test and histopathology to PCR is shown in Table 4.

*H. heilmannii* sensu stricto was detected in 38 dogs (63.3%), including mixed infections in 24 dogs, followed by *H. bizzozeronii* in 32 dogs (53.3%), including mixed infections in 23 dogs, *H. salomonis* in 4 dogs (6.7%), *H. felis* in 1 dog (1.7%), including mixed infection in 1 dog and undetermined *Helicobacter* species in 8 dogs (13.3%). Mixed infections were detected in 24 dogs (40%) with positive PCR assay. No *H. pylori* was found in any of the dogs. Prevalence of different *Helicobacter* species and associated histopathologic findings is given in Table 5.

In 22 (33.8%) positive and 6 (31.6%) negative dogs the endoscopic appearance of the gastric mucosa was normal. Overall, the most common endoscopic lesions were increased granularity of gastric mucosa and erosions or ulcers. Complete description of endoscopic findings is summarized in Table 6.

There was no correlation between *Helicobacter* infection status and any of the endoscopic findings. Gastritis was diagnosed histologically in 38.5% (25 of 65) of dogs positive and in 47.4% (9 of 19) of dogs negative for *Helicobacter* spp. Histologic gastritis score in dogs with or without *Helicobacter* infection is presented in Table 6. Most often, the bacteria were observed in the lumen of gastric glands or in the superficial part of the gastric mucus.

There was no association between *Helicobacter* infection status and the presence of gastritis and inflammatory infiltration (*p* = 0.30), epithelial injury (*p* = 0.69) or fibrosis (*p* = 1). Gastric lymphoid follicular hyperplasia was detected more frequently and was more extensive in *Helicobacter* infected than non-infected dogs (*p* = 0.034).

There was a significant difference in the degree of inflammation of dogs with single compared to mixed *Helicobacter* infection (*p* = 0.003). *Helicobacter* mono-infection was associated with 2.4 times increased odds of having more severe inflammation (*p* = 0.012).

## 4. Discussion

This study identified the prevalence of individual *Helicobacter* species and assessed their association with histologically confirmed gastritis in a large sample of dogs presented with chronic GI clinical signs. A significantly higher degree of inflammation in dogs with single compared to mixed *Helicobacter* infection was detected. Gastric lymphoid follicular hyperplasia was more frequently associated and was more extensive in *Helicobacter* infected than non-infected dogs. Although not statistically significant, common endoscopic lesions were erosions or ulcers. Diagnostic value of invasive tests was determined.

Using a combination of diagnostic tests, 77.4% dogs in this cohort were *Helicobacter* positive. Similar prevalence (74–95%) was reported in different groups of dogs with GI signs [33,34,35]. In dogs from random sources (with or without GI signs), *Helicobacter* was detected in 77–92% [10,24,36,37,38,39]. In healthy dogs (privately owned or laboratory dogs), the prevalence of gastric *Helicobacter* infection was 67–100% [1,21,40,41,42,43].

*H. heilmannii* sensu stricto was the predominant *Helicobacter* species in this study. This species was also frequently documented in other studies [21,24,33,39,44]. However, due to changes in the taxonomy of *Helicobacter* spp., it is difficult to compare the prevalence of *H. heilmannii* sensu stricto in this study with data from older studies in which a positive result for *H. heilmannii* may have included species such as *H. felis*, *H. bizzozeronii*, or *H. salomonis* in addition to *H. heilmannii* sensu stricto. In addition, geographic differences in the occurrence of NHPH species have been documented [24,44].

The second most prevalent *Helicobacter* in this study was *H. bizzozeronii*, which is generally considered the prevailing NHPH species in dogs [13,14,15,20,23,24,33]. It was identified in 55.6% of canine gastric biopsies [14], in 65% of infected dogs [33] and 70% of canine gastric samples (20% as single, 50% as mixed infections) [24]. *H. bizzozeronii*–like organisms was reported as the predominant species in colony-raised Beagle dogs [40].

*H. salomonis* has only sporadically been detected in dogs (8–9%) [15,24]. More than 50% of investigated Portuguese dogs harbored *H. salomonis*, mostly in mixed infections [39]. This species was the only *Helicobacter* sp. not found in mixed infections in this study.

Using PCR, *H. felis* was found in 4.8% of examined dogs [38], in 8.7% and 11.9% of infected dogs [33,45] and isolated from 22.2% of gastric biopsies [14]. More than 50% of the investigated Belgian dogs harboured *H. felis*, mostly in mixed infections [24].

In 13.3% of *Helicobacter*-positive dogs, the species remained undetermined. We have not attempted to demonstrate the recently described species *H. cynogastricus* or “*H. rappini*” and *H. bilis* that were previously isolated from the canine stomach [42,46].

No *H. pylori* was found in dogs examined in the study reported here. According to overwhelming majority of studies, *H. pylori*, the most prevalent species in humans, is virtually absent in dogs and thus it is unlikely that dogs play an important role in the transmission of this organism to humans. To our knowledge, there are only a few reports of *H. pylori* infection in dogs [29,44,47,48,49].

It is now a well-known fact that different *Helicobacter* species co-inhabit the same regions of the gastric mucosa [23]. Mixed infections were identified in gastric samples from 16.7% to 48.5% of dogs [24,33,39] and the fundic mucosa was co-infected with *H. bizzozeronii* and *H. felis* in all asymptomatic Beagle dogs [20]. This high rate of mixed infections detected in canine stomachs is in contrast to their low prevalence in human samples (16.3%) [24]. In the present study, a correlation between the presence of mixed infection (including *H. heilmannii* and *H. bizzozeronii*) and the lower degree of gastric inflammation compared to mono-infection was detected. Competitive inhibition may occur in gastric helicobacteriosis; the infection by one organism suppresses proliferation of other helicobacters. This phenomenon has been suggested as a reason for the rare occurrence of concurrent infection with NHPH and *H. pylori* in humans and nonhuman primates [50,51]. *H. heilmannii* infection might protect from infection with *H. pylori* [52]. Furthermore, when puppies with gastric *H. salomonis* were experimentally infected with *H. bizzozeronii*, infection by *H. salomonis* was suppressed [53].

Different *Helicobacter* species or even strains differ in their virulence factors and pathogenicity [10,15,22]. Due to taxonomic inconsistency and diagnostic difficulties, the majority of earlier studies usually did not discriminate between individual NHPH species. Notable exceptions are represented by several descriptions of a link between experimental or natural infection with certain NHPH species (now separated from the former “*H. heilmannii*”) and gastric pathology [5,6,22,33,37]. The most pronounced histopathologic lesions in the present study were found in association with *H. bizzozeronii* and *H. heilmannii* sensu stricto. The findings include severe LPG (11.1%) and acute purulent-necrotic gastritis in single *H. bizzozeronii* infections and moderate LPG (14.3%) in single *H. heilmannii* sensu stricto infection. *H. bizzozeronii* induced mild to moderate lymphocytic and neutrophilic infiltration in the gastric antrum of some Mongolian gerbils, which was sometimes accompanied by parietal cell loss [54]. Overall, *H. bizzozeronii* appears to be more host-adapted in dogs and associated with a lower pathogenicity than *H. pylori* or *H. felis* [20,55,56]. Infection with nine different *H. heilmannii* sensu stricto isolates in the Mongolian gerbil model showed that strains had different abilities to colonize the stomach. Furthermore, 78% of the strains induced chronic active gastritis and lymphocytic aggregation [22]. Overall, these studies demonstrate that not only are there differences in the bacterium-host interactions between diverse NHPH species, but there are also differences in the pathogenic potential in strains within the same species. Further studies are necessary to determine the virulence factors involved and their putative associations with disease [57].

Numerous detection methods for the presence of *Helicobacter* spp. have been developed. Each one has advantages and disadvantages [58]. There is a need for a reference method to be used as “gold diagnostic method.” Unfortunately, none of the currently used methods can meet this criterion. PCR based diagnosis may be considered as gold standard by designing primers specific to target genes-the approach used in this study. Another solution is to combine the results of two or more techniques and compare with results of each method being evaluated [58].

It is not possible to differentiate individual NHPH species by traditional bacteriological methods. In addition, it is also difficult using molecular techniques, since there is a high interspecies similarity in gene sequences which can lead to confusion between closely related species [9,59,60]. On the other hand, the genetic diversity within certain species can be very high [61]. PCR-based techniques are the preferred method for conclusive *Helicobacter* species identification [24,62]. However, the choice of the target gene is of major importance [6]. For example, 16S rDNA-based PCR assay targeting a 78-bp DNA fragment cannot discriminate between *H. bizzozeronii*, *H. salomonis* and *H. felis*, but detects these species as a group [27]. However, tests based on detection or sequencing of the hsp60 gene, the urease A and B genes or gyrB gene allow identification of NHPH to the species level [5]. For three dogs (3.6%) negative by all other diagnostic tests, PCR results were positive. This suggests that the degree of colonization might be too low to be detected by microscopy-based techniques. For three dogs (3.6%) positive by cytology and/or histologic examination, PCR results were negative. Perhaps these negative results were due to the patchy distribution of organisms within the stomach or *Helicobacter* spp. whose DNA our primers were unable to amplify.

In earlier studies, cytology was found to be reliable for detecting *Helicobacter* infection in dogs. It was more sensitive than histologic examination or the rapid urease test and can be therefore considered the method of choice for demonstrating NHPH [12,15,37]. However, the extent and intensity of concurrent gastritis cannot be evaluated [63]. Because of the patchy distribution of organisms within the stomach, examination of samples from multiple locations in the corpus and fundus increases sensitivity [64]. For exact species determination, cytology is unreliable [9,14,42,64,65,66].

Histopathologic examination is considered less sensitive than cytology; only 66% of stomachs were *Helicobacter*-positive on histopathology compared to 84% positivity of direct microscopic examination [37]. Its sensitivity (92%) is somewhat better than that of the urease test (85–87%) [12] or worse [37], based on selected literature. In this study, histology had the lowest sensitivity and specificity of all methods used. Helicobacters associated with the mucosal surface or localized within gastric pits are relatively easy to detect with routine HE staining of tissues [64]. However, bacteria in gastric glands and glandular epithelial cells should be more readily detected with a modified silver stain. The WS technique should be significantly more sensitive than HE staining [67]. Although some authors claim that silver stains are needed to identify these organisms, others state that the WS technique revealed NHPH in only one of the samples in which HE staining had failed to detect them [12]. In this study, both staining techniques yielded identical results. It may also be important to mention that organisms could be lost in the process of fixation/paraffin embedding.

The urease test is generally less sensitive than cytology; it was positive in 72% of examined canine stomachs compared to 84% positivity of direct microscopic observation [37]. It is also less sensitive than *Helicobacter* genus-specific PCR assay and histology [33,38,62]. Compared to PCR as a reference technique, the urease test had a sensitivity of 86.4% and a specificity of 66.7% [33]. This sensitivity of the rapid urease test is comparable with data in the present study; however, specificity in this study is substantially higher. In 12 dogs in this study (14.3%) the urease test was negative while some or all other tests yielded positive results. False negative results can be caused by the patchy distribution of bacteria within the stomach, bleeding or the use of drugs that decrease acid secretion, since increase in pH alters the activity of urease [26,58,64]. Negative results can also be associated with a small number of helicobacters in the sample [37] or with rare urease-negative *Helicobacter* spp. [58,63]. When the NHPH-count is low, the number of positive results increases with the time after which the test is read [12,16]. In one dog of 84 (1.2%) only the urease test was positive. A possible false positive cannot be excluded. Other bacteria which can occur in the stomach such as *Proteus mirabilis* or *Pseudomonas aeruginosa* produce urease that can lead to a false positive test result for *Helicobacter* spp. [12]. The urease test does not give any information about the species identity.

Direct association between *Helicobacter* colonization and the degree of gastric pathology was not found in the present study. This is in agreement with previous studies in which gastritis was not directly linked to the degree of colonization and no significant association was detected between the intensity of *Helicobacter* infection and degree of gastric inflammation [15,33,37,39,41,42,43,68,69]. In dogs with *Helicobacter* infection, normal gastric histologic findings were recorded for more clinically normal animals than for those with chronic vomiting [15].

Gastric NHPH infections in humans can cause chronic active gastritis with mainly focal, less severe lesions than those seen during *H. pylori* infection. Although NHPH are less frequently associated with erosions, ulceration, or neoplasia [70,71], infection with NHPH in humans has been associated with gastritis, peptic ulcer disease and mucosa associated lymphoid tissue (MALT) lymphoma [5,11,22,24,26,28,59,72]. In dogs, pathologic changes linked with the NHPH infection are generally even milder than those observed in humans. Therefore, the finding of erosions/ulcers as frequent endoscopic lesions, and frequently encountered microscopic epithelial injury and purulent-necrotic gastritis in *Helicobacter*-positive dogs encountered in this study was somewhat surprising. Although GI ulcers or neoplasia generally have not been associated with *Helicobacter* infection in dogs, and no association has been made between *Helicobacter* infection and GI ulcers [73] and erosions (they are found only rarely [74]), mucosal defects ranging from small erosions to ulceration have been reported in 2.2% of asymptomatic infected experimental Beagle dogs [40].

Gastric inflammation in NHPH infection is generally mononuclear in nature and ranges from mild to moderate in severity [20,21,34,42]. Gastric lymphoid follicular hyperplasia accompanies infection in some but not all animals [1,15,16,73,74]. Gastric lymphoid hyperplasia appears to be more common and more extensive in *Helicobacter* infected than uninfected dogs and cats [73,75], which is also supported by this study. Although fibrosis was found in 45–59% of examined canine stomachs, it did not seem to be associated with the presence of helicobacters [16]. No association between the presence of fibrosis and *Helicobacter* infection was found in the present study.

The high prevalence of gastric infection with NHPH in healthy and sick dogs indicates that there is no simple “infection-disease” relationship in dogs. Thus, it may be inferred that *Helicobacter* spp. are not pathogenic in dogs and are more commensals than pathogens. Therefore, the development of gastritis and vomiting in some infected dogs can perhaps be attributed to the loss of tolerance to gastric NHPH rather than to their pathogenicity. Using a mouse *H. pylori*-infection model, it has been reported that the development of tolerance to *H. pylori* protects from gastric cancer precursor lesions. Thus, different susceptibility of infected individuals to *H. pylori* might be associated with variable disease manifestations [76]. It has also been reported that despite a high level of colonization and ultrastructural findings, no clinical signs and only mild histological gastritis were observed in research colony dogs, suggesting that immune tolerance might be involved in canine *Helicobacter* spp. infection [20]. However, this view may be somewhat inaccurate as prevalence of *H. pylori* in humans is over 80% in some countries, but only a relatively small number (15–20%) of them have overt clinical signs of infection [4,73]. Additionally, although *H. pylori* is a well-adapted and highly abundant resident of the gastric environment in people, if present it is not alone. At least 262 different phylotypes have been identified in the human stomach [75,77,78,79], though *H. pylori* is dramatically more abundant than other members of the community, accounting for up to 97% of all sequences [75,77]. Also, *H. pylori* infection has been associated with distinct gastric microbial community structures [80], which may in part determine the outcome of *H. pylori* infection.

## 5. Conclusions

In conclusion, dogs with chronic gastrointestinal signs are frequently infected by helicobacters other than *H. pylori*. PCR with suitable primers is the method of choice for detection, and that can differentiate between individual *Helicobacter* species. *H. heilmannii* sensu stricto was the predominant *Helicobacter* species in this study. Cytology and rapid urease test performed on gastric biopsy samples may be reliable diagnostic methods suitable for clinical practice. *Helicobacter* mono-infection was associated with an increased risk of having more severe inflammation. An association between infection and lymphoid follicular hyperplasia was demonstrated. NHPH infection should be considered in cases of gastric erosions and ulcers. An association between *Helicobacter* infection status and presence of gastritis was not found. NHPH species colonizing stomach of dogs and their zoonotic potential certainly deserve further research attention.

## Figures and Tables

**Table 1:** Primer sequences for Helicobacter genus and species-specific PCR amplification used in the study of 84 dogs with chronic gastrointestinal signs.

Species	Primer Sequence and Direction (5′→3′)		Gene Target	GenInfo Identifier	Amplified Fragment Size (bp)
*Helicobacter* spp.	Hspp-F	TACACCAAGAATTCCACCTA	16S ribosomal RNA gene	Gi:373809908	259
	Hspp-R	CGTGGAGGATGAAGGTTTTA			
*Helicobacter pylori*	HP1-F	ATGAAAAAGATTAGCAGAAAAG	*H. pylori* urease (*ureA*, *ureB*, *ureC*, *ureD*) genes	Gi:15644634	1668
	HP1-R	CCTAGAAAATGCTAAAGAGTTG			
	HP2-F	GCGGCTGAAGAATATTCTATGA			689
	HP2-R	CGCTGGGTTAATGGTGTATTTAG			
*Helicobacter felis*	HF1-F	ATGAAACTAACGCCTAAAGAACTAG	*H. felis* urease (*ureA* and *ureB)* genes	Gi:396160	1147
	HF1-R	GGAGAGATAAAGTGAATATGCGT			
	HF2-F	CTCATTAGCGGGCGTGTGAT			891
	HF2-R	CAATCTTGCCGTCTTTAATCCC			
*Helicobacter heilmannii* sensu stricto ASB1.4	HH-F	TACACCAAGAATTCCACCTA	*H. heilmannii* sensu stricto urease (*ureA* and *ureB*) genes	Gi:408906187	220
	HH-R	AATTCCACCTACCTCTCCC			
*Helicobacter salomonis*	HS-F	TGCGTAGGCGGGGTTGTAAG	16S ribosomal RNA	Gi:1915896	75
	HS-R	CAGAGTTGTAGTTTCAAATGC			
*Helicobacter bizzozeronii*	HB-F	AACCAACAGCCCCAGCAGCC	*H. bizzozeronii* urease (*ureA* and *ureB)* genes	Gi:27462193	373
	HB-R	TGGTTTTAAGGTTCCAGCGC			

F, forward; R, reverse.

**Table 2:** Demographic data of 84 dogs with chronic gastrointestinal signs enrolled into the study.

	*Helicobacter* Positive	*Helicobacter* Negative
Number of included dogs	65	19
Mean age	5.4 ± 3.7 years	5.9 ± 4.1 years
Age range	6 months–13 years	6 months–13 years
Mean body weight	20.9 ± 14.3 kg	19.1 ± 13.9 kg
Body weight range	3.2–59 kg	2.8–57 kg
Sex	38 males (1 neutered)	9 males (1 neutered)
	27 females (4 spayed)	10 females (4 spayed)
The most common breeds	Yorkshire Terrier (6 dogs), German Shepherd (5 dogs), Boxer, Golden Retriever (each 4 dogs), Rhodesian Ridgeback, Bullterrier, Mixed breed, West Highland White Terrier (each 3 dogs), Border Collie, Borzoi, Jack Russell Terrier, Labrador Retriever (each 2 dogs)	Yorkshire Terrier, Mix breed (each 3 dogs) Maltese and Vizsla (each 2 dogs)

**Table 3:** Concordance of test results for *Helicobacter* infection in 84 dogs with chronic gastrointestinal signs.

Combination Pattern	Rapid Urease Test	Cytology	Histopathology (Both HE and WS)	PCR	No. of Dogs with Pattern in the Cohort of 84 Dogs (%)
1.	+	+	+	+	42 (50%)
2.	−	−	−	−	19 (22.6%)
3.	+	+	−	+	5 (6%)
4.	−	+	+	+	4 (4.8%)
5.	+	−	+	+	3 (3.6%)
6.	−	−	−	+	3 (3.6%)
7.	+	−	+	−	2 (2.4%)
8.	−	+	−	+	2 (2.4%)
9.	−	+	+	−	1 (1.2%)
10.	−	+	−	−	1 (1.2%)
11.	+	−	−	+	1 (1.2%)
12.	−	−	+	−	1 (1.2%)

HE, hematoxylin, and eosin; WS, Warthin-Starry stain; +, positive; −, negative.

**Table 4:** Comparison of the diagnostic value of cytology, rapid urease test and histopathology to PCR in the study of 84 dogs with chronic gastrointestinal signs.

Test	Sensitivity	Specificity	Positive Predictive Value	Negative Predictive Value
Cytology	88.3%	91.7%	96.4%	81.5%
Rapid urease test	85%	91.7%	96.2%	71%
Histopathology HE	81.7%	83.3%	92.5%	64.5%
Histopathology WS	81.7%	83.3%	92.5%	64.5%

**Table 5:** Histopathologic findings in 60 dogs infected with different *Helicobacter* species (detected by PCR).

	LPG (Mild)	LPG (Moderate)	LPG (Severe)	Neoplasia	Nonspecific Changes	Acute Purulent-Necrotic Gastritis	No. of Dogs with Result among 60 PCR Positive Dogs (%)
*Helicobacter heilmannii* sensu stricto	6/14	2/14	-	1/14	5/14	-	14 (23.3%)
*Helicobacter bizzozeronii*	6/9	-	1/9	-	1/9	1/9	9 (15.0%)
*Helicobacter salomonis*	-	-	-	-	3/4	1/4	4 (6.7%)
*Helicobacter felis*	-	-	-	-	1/1	-	1 (1.7%)
*Helicobacter heilmannii* sensu stricto + *Helicobacter bizzozeronii*	1/23	2/23	-	2/23	18/23	-	23 (38.3%)
*Helicobacter heilmannii* sensu stricto + *Helicobacter felis*	-	-	-	-	1/1	-	1 (1.7%)
*Helicobacter*-undetermined species	2/8	-	-	-	6/8	-	8 (13.3%)
No. of dogs with result (% of 60 PCR-positive dogs)	15 (25.0%)	4 (6.7%)	1 (1.7%)	3 (5.0)	35 (58.3%)	2 (3.3%)	60 (100%)

LPG, lymphocytic-plasmacytic gastritis.

**Table 6:** Results of histopathology and endoscopic findings in 65 *Helicobacter* positive dogs (using a combination of diagnostic tests for *Helicobacter* spp. including rapid urease test, cytology, histopathology, and PCR) and 19 dogs negative for *Helicobacter* spp.

	*Helicobacter* Positive	*Helicobacter* Negative
Histopathological description		
LPG +	18 (27.7%)	4 (21.1%)
LPG ++	4 (6.2%)	2 (10.5%)
LPG +++	1 (1.5%)	1 (5.3%)
acute purulent gastritis	2 (3.1%)	-
eosinophilic gastritis	-	2 (10.5%)
epithelial injury	58 (89.2%)	16 (84.2%)
fibrosis	18 (27.7%)	5 (26.3%)
lymphoid follicular hyperplasia	28 (43.1%)	3 (15.8%)
non-specific	37 (56.9%)	8 (42.1%)
neoplasia	3 (4.6%)	2 (10.5%)
	(*lymphoma, gastric adenocarcinoma, GI stromal tumor*)	(*gastric adenocarcinoma, leiomyoma*)
Endoscopic findings		
difficult to inflate lumen	4 (6.15%)	1 (5.3%)
edema	4 (6.2%)	1 (5.3%)
erosion/ulcer	16/3 (29.2%)	2/3 (26.3%)
friability	3 (4.6%)	1 (5.3%)
granularity	29 (44.6%)	10 (52.6%)
hyperemia	5 (7.7%)	1 (5.3%)
hypertrophy	1 (1.5%)	2 (10.5%)
irregularity	1 (1.5%)	1 (5.3%)
mass	1 (1.5%)	1 (5.3%)
normal finding	22 (33.8%)	6 (31.6%)
polyp	2 (3.1%)	3 (15.8%)

Nonspecific changes-mild edema and hyperemia of lamina propria; LPG, lymphocytic-plasmacytic gastritis.

## Data Availability

Not applicable.

## Supplementary Materials

The following supporting information can be downloaded at: https://www.mdpi.com/article/10.3390/ani12101254/s1.
