# Supplementary material for: Helicobacter Species and Their Association with Gastric Pathology in a Cohort of Dogs with Chronic Gastrointestinal Signs
Source: Animals (Basel). 2022 May 13;12(10):1254. doi: 10.3390/ani12101254 (PMC9137851; doi:10.3390/ani12101254)
Supplement: Supplementary file 1 [file animals-12-01254-s001.zip › animals-1708254-supplementary.pdf]

Supplementary File S1. The PCR amplification parameters for detection of individual *Helicobacter* species.

Detection of *Helicobacter* spp.

The PCR detected the 16S ribosomal RNA gene sequence. The reaction using Hspp-F and Hspp-R primers included primary incubation at 95˚C for 15 minutes, 3 cycles of denaturation at 95˚C for 60 s, annealing at 58˚C for 60 s, and elongation at 72˚C for 60 s, followed by 34 cycles of denaturation at 95˚C for 60 s, annealing at 58˚C for 30 s and elongation at 72˚C for 30 s.

Detection of *Helicobacter pylori*

The PCR detected the *H. pylori*-specific urease gene sequence. Two subsequent PCR amplifications were performed. PCR with primers HP1-F and HP1-R consisted of primary incubation at 95˚C for 15 minutes, 3 cycles of denaturation at 95˚C for 60 s, annealing at 57˚C for 120 s, elongation at 72˚C for 60 s followed by 35 cycles of denaturation at 95˚C for 60 s, annealing at 62˚C for 30 s and elongation at 72˚C for 60 s. Amplification product was used as a template for the second PCR with HP2-F and HP2-R primers consisting of primary incubation at 95˚C for 15 minutes, 3 cycles of denaturation at 95˚C for 60 s, annealing at 57˚C for 120 s, and elongation at 72˚C for 60 s, followed by 35 cycles of denaturation at 95˚C for 60 s, annealing at 67˚C for 30 s and elongation at 72˚C for 60 s.

Detection of *Helicobacter felis*

The PCR detected the *H. felis*-specific urease gene sequence. Two PCR amplifications were performed. PCR with primers HF1-F and HF1-R consisted of primary incubation at 95˚C for 15 minutes, followed by 35 cycles of denaturation at 95˚C for 60 s, annealing at 62˚C for 30 s and elongation at 72˚C for 60 s. Amplification product was used as a template for the second PCR with HP2-F and HP2-R primers consisting of incubation at 95˚C for 5 minutes, followed by 3 cycles of denaturation at 95˚C for 60 s, annealing at 57˚C for 120 s, elongation at 72˚C for 60 s followed by 35 cycles at 95˚C for 60 s, annealing at 67˚C for 30s and elongation at 72˚C for 60s.

Detection of *Helicobacter heilmannii* sensu stricto

The PCR detected the *H. heilmannii* sensu stricto-specific urease gene sequence. PCR with primers HH1-F, HH1-R and HH2-F, HH2-R consisted of primary incubation at 95˚C for 15 minutes, annealing at 57˚C for 120 s and elongation at 72˚C for 60 s followed by 35 cycles of denaturation at 95˚C for 60 s, annealing at 67˚C for 30 s and elongation at 72˚C for 60 s.

Detection of *Helicobacter salomonis*

The PCR detected the 16S ribosomal RNA gene sequence. PCR with primers HS-F and HS-R consisted of primary incubation at 95˚C for 15 minutes, followed by 35 cycles of denaturation at 95˚C for 60 s, annealing at 63˚C for 60 s and elongation at 72˚C for 60 s.

Detection of *Helicobacter bizzozeronii*

The PCR detected the *H. bizzozeronii*-specific urease gene sequence. PCR with HB1-F and HB1-R primers included primary incubation at 95˚C for 15 minutes, 3 cycles of denaturation at 95˚C for 60 s, annealing at 58˚C for 60 s, and elongation at 72˚C for 60 s, followed by 35 cycles of denaturation at 95˚C for 60 s, annealing at 63˚C for 60 s and elongation at 72˚C for 60 s.
